# Plant species-specific rhizobiome assembly in the hyper-arid Atacama Desert

**DOI:** 10.3389/fmicb.2025.1587491

**Published:** 2025-09-16

**Authors:** J. Fortt, J. Castro-Severyn, A. Choque, G. Donoso, A. Stoll, D. L. Jones, C. P. Saavedra, B. Fuentes, F. Remonsellez

**Affiliations:** ^1^Departamento de Ingeniería Química y Medio Ambiente, Universidad Católica del Norte, Antofagasta, Chile; ^2^Centro de Investigación Tecnológica del Agua y Sustentabilidad en el Desierto (CEITZASA), Universidad Católica del Norte, Antofagasta, Chile; ^3^Centro de Estudios Avanzados en Zonas Áridas (CEAZA), La Serena, Chile; ^4^School of Environmental and Natural Sciences, Bangor University, Bangor, Gwynedd, United Kingdom; ^5^Laboratorio de Microbiología Molecular, Facultad de Ciencias de la Vida, Universidad Andrés Bello, Santiago, Chile

**Keywords:** rhizosphere, *Suaeda foliosa*, *Distichlis spicata*, microbial community, hyper-arid core

## Abstract

The hyper-arid core of the Atacama Desert represents one of the oldest and driest regions of the world and is characterized by high aridity (precipitation <2 mm y^−1^), hypersaline soil conditions, extremes in temperature (−5 °C to 50 °C), intense UV irradiation and low organic matter content. Despite this, the Yungay area within the hyper-arid core is capable of supporting vegetation adapted to these extreme environmental conditions, including *Distichlis spicata* and *Suaeda foliosa*, which access deep groundwater resources. Little is known, however, about the below-ground microbial community that these plants support. To understand plant-microbe interactions in this environment, we investigated the physicochemical properties in the rhizosphere soils of *D. spicata* and *S. foliosa*. In addition, DNA was extracted from the rhizosphere soil and 16S rRNA gene amplicon sequencing performed to describe the taxonomic composition of the bacterial community. Our results revealed significant differences in the physicochemical properties between the rhizosphere soils of the two native plants. *D. spicata* showed higher Electrical Conductivity (EC), while *S. foliosa* had elevated ammonium concentrations. The microbial composition also varied between the plant species: Firmicutes (Bacillota), Proteobacteria (Pseudomonadota), Halobacteria, and Actinobacteriota (Actinomycetota) were dominant in both plant rhizosphere samples, but their relative abundance differed. In this context, Halobacteria were highly represented in the soils of *D. spicata* and Firmicutes (Bacillota) in those from *S. foliosa*. Furthermore, bacterial genera such as *Enterococcus* were only present in the *S. foliosa* rhizosphere, while *Natrinema* was highly represented in soil from under *D. spicata* (33.4%) in comparison to *S. foliosa* (1.5%). The microbial community of *D. spicata* was strongly influenced by EC, whereas that of *S. foliosa* correlated more with ammonium levels. These findings advance our understanding of microbial community adaptation in one of Earth’s most extreme environments and provide new insights into plant-microbe interaction in hyper-arid soils.

## Introduction

The Atacama Desert, one of the driest and oldest deserts on Earth, extends from 18°S to 27°S and is characterized by extreme environmental conditions including minimal precipitation, high soil salinity, and intense solar radiation (Bull et al., 2016). This desert, particularly its hyper-arid core, experiences an aridity index (AI) of < 0.03 with mean annual rainfall below 2 mm, and temperatures fluctuating drastically between day and night (Azua-Bustos et al., 2017; Hartley et al., 2005; Lacap et al., 2011; Ritter et al., 2018). The Yungay zone, located within this hyper-arid core, presents a Mars-like surface and has remained one of the most extreme environments for millions of years (Clarke, 2006; Hartley et al., 2005; Mckay et al., 2003; Navarro-González et al., 2003). The prevailing environmental conditions create significant abiotic stress, limiting the establishment of vegetation and promoting the accumulation of salts in the soil (e.g., NaCl, KNO_3_, NaClO_4_, CaSO_4_), which stratify due to the absence of leaching (Azua-Bustos et al., 2017; Bull et al., 2016; Voigt et al., 2020; Wang et al., 2017). Despite the poly-extreme conditions prevailing in hyper-arid environments, many studies have reported an abundance of life, implying that biogeochemical recycling of key nutrients (e.g., C, N and P) does occur (Fuentes et al., 2022a; Fuentes et al., 2022b; Knief et al., 2020). In this regard, several studies have reported that plants and associated microbial communities in the Atacama Desert both participate in soil nutrient cycling and the rapid transport of carbon (C) and nitrogen (N), particularly in response to ephemeral inputs of moisture (Jones et al., 2018a; Jones et al., 2018b; Santander et al., 2021; Wu et al., 2021). Interestingly, in the Yungay zone it is possible to find an oasis called the “Yungay Oasis,” which supports a high density of plants and shrubs (Fletcher et al., 2012). Here it is possible to find patches of the halophytes *Distichlis spicata* and *Suaeda foliosa* (Calderon et al., 2015). These plants obtain moisture from superficial groundwater, specifically they receive water from an aqueduct that was made for a mineral processing plant that was abandoned over 80 years ago (Chávez et al., 2016; Fletcher et al., 2012; Garrido et al., 2016).

Microbial life in hyper-arid deserts like the Atacama has evolved to withstand extreme environmental pressures, playing crucial roles in nutrient cycling and ecosystem stability (Bull and Asenjo, 2013). Previous studies have shown that even in the most extreme arid soils, microbial communities facilitate the rapid turnover of C and N, especially in response to rare moisture inputs (Ewing et al., 2008; Knief et al., 2020). Several works indicate that is possible to find a great biodiversity, which includes hypolithic cyanobacteria (Warren-Rhodes et al., 2006), non-lichenized fungi (Conley et al., 2006; Fuentes et al., 2020), lichens (Rundel, 1978), cacti (Rundel et al., 1991), and even shrubs and trees (Fletcher et al., 2012), which are unevenly distributed and their development is pressured by biotic and abiotic factors, such as plant disease, biocontrol, topography and hydrology, respectively (Lin et al., 2011; Poulos and Camp, 2010). This microbial diversity, ranging from hypolithic cyanobacteria to fungi, lichens, and bacteria, thrives despite the scarcity of organic matter and water (Wu et al., 2021). In this context, the microbial processes in such environments are critical for sustaining the sparse vegetation and are particularly sensitive to any changes in moisture availability (Pointing and Belnap, 2012). Understanding the microbial diversity and its functional roles in these ecosystems therefore provides unique insights into the resilience and adaptability of life under extreme abiotic stress (Makhalanyane et al., 2015).

In desert ecosystems, the plant–soil-microbial interface (i.e., the rhizosphere) serves as a biological hotspot where plants engage in complex symbiotic relationships with diverse microbial communities (Bonfante and Anca, 2009; Dhanker et al., 2020). These rhizobiomes are essential for nutrient acquisition and stress tolerance, helping native plants from arid zones survive in environments characterized by high salinity and drought (Kim et al., 2013; Niu et al., 2018). In this context, the symbiotic relationships in the rhizosphere can modulate plant metabolism through the production of phytohormones, enhancement of osmoregulatory mechanisms, and increased photosynthetic efficiency (Dhanker et al., 2020; Ruiz-Sánchez et al., 2010). These interactions are particularly relevant in the Atacama Desert, where plants rely on microbial consortia to mitigate the harsh abiotic conditions (Neilson et al., 2012; Stotz et al., 2021). Hence, research on rhizobiomes in extreme environments such as the Atacama Desert, is of critical importance as these microbial communities play a key role in enhancing plant resilience to abiotic stressors (Bashan et al., 2014; Egamberdieva et al., 2017), and understanding these plant-bacteria interactions has the potential to uncover mechanisms of plant resilience and offer strategies for improving agricultural productivity in arid regions (Richardson et al., 2009; Vurukonda et al., 2016).

According to the unique environmental conditions of the Yungay Oasis and the known role of microbial communities in supporting plant life under extreme stress, this study aims to investigate the rhizospheric microbial communities of the main two native plants colonizing the area, namely *Suaeda foliosa* and *Distichlis spicata*. Specifically, we seek to characterize the physicochemical parameters of their rhizospheric soils and correlate these with the taxonomic composition of the bacterial communities. By analyzing the relationship between abiotic soil conditions and microbial diversity, we aim to determine how the hyper-arid soil conditions of the Yungay Oasis in the Atacama Desert modulate rhizosphere microbiomes. This study will contribute to a deeper understanding of the plant-bacteria interactions that enable survival in one of the most extreme environments on Earth, with potential applications for enhancing crop resilience in the north of Chile and similar arid regions.

## Methodology

### Sampling site description

**Figure 1:**
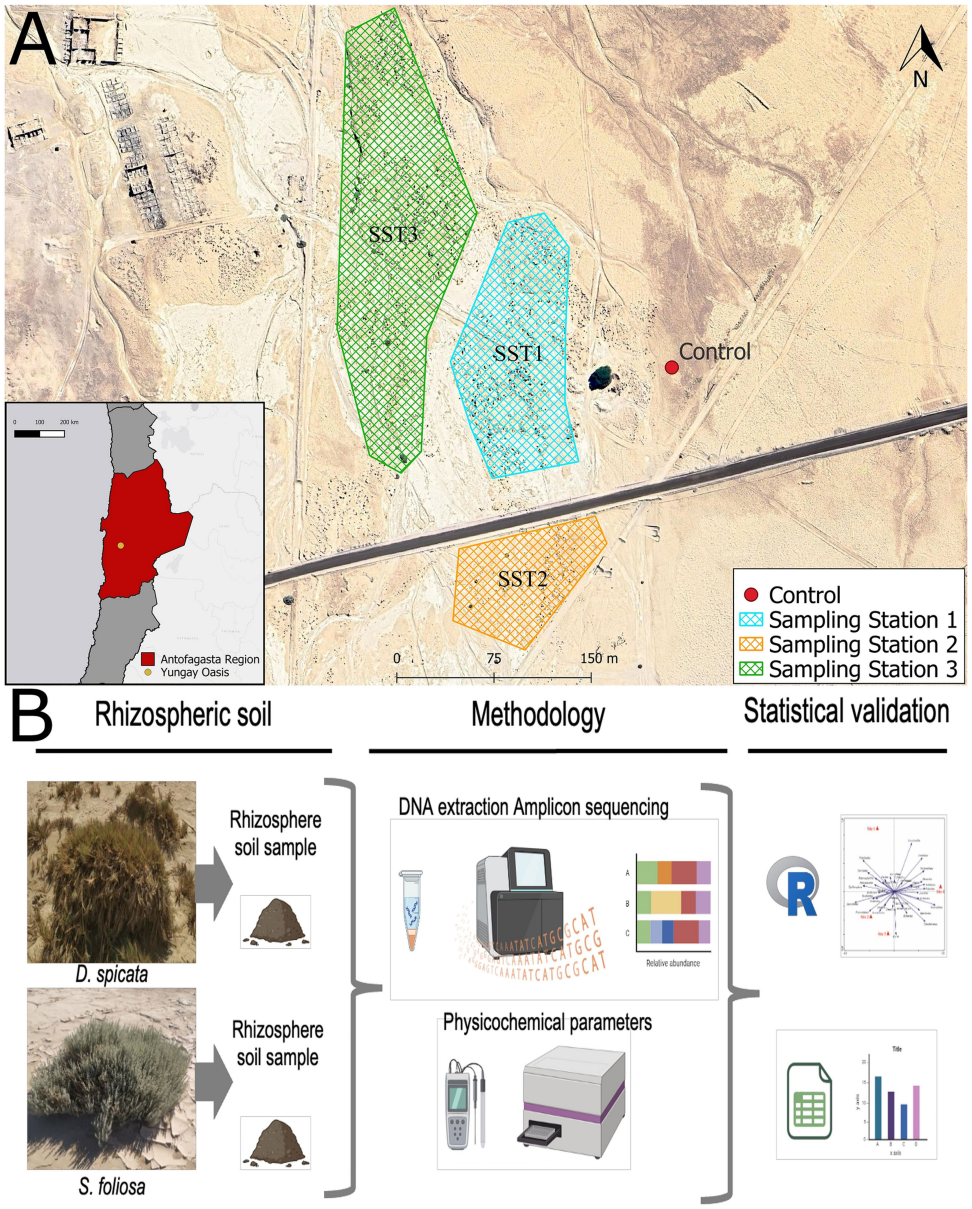
**(A)** Study area location and the three sampling stations (SST1-SST3). **(B)** Photographs of the plant species from the Yungay Oasis and an overview of the main methodological processes used in the study.

The study site was located within the Yungay area, in the hyper-arid core of the Atacama Desert in the Antofagasta region of Chile (24°03′S, 69°49′W; 948 meters above sea level; Jones et al., 2023). This study was focused on the rhizosphere soil of the native plants, namely the drought tolerant shrub *Suaeda foliosa* Moq. (de Froidmont, 2018) and the salt grass *Distichlis spicata* (L.) Greene (Pelliza et al., 2005). We selected three sampling station zones covering approximately 2,500 m^2^. Rhizosphere soil was collected in 50 mL falcon tubes and sterile bags from five individuals per species and three control samples corresponding to bare soil (Figure 1A). Both kinds of samples (rhizosphere and control soil) were collected from 0–20 cm depth. A total of 33 soil samples were taken specifically from the Yungay Oasis (Sampling Stations 1, 2 and 3), 30 samples from the plant rhizosphere and 3 control (unvegetated bare soil) samples. A part of each sample was stored in fresh and dry conditions at room temperature (20–25 °C) for physicochemical analysis, while the other part was stored at −80 °C for followed DNA extraction.

### Soil physicochemical characterization

All samples were taken and processed as shown in Figure 1B and taken to the laboratory to conduct physicochemical, microbiological and molecular procedures. The moisture content (MC) of the soil sample was measured by drying soil at 105 °C in the oven and calculating the loss of weight after drying. Furthermore, the EC and pH were measured using standard electrodes in a soil-distilled water suspension with a solution-to-soil ratio of 1:2.5 (w/v). The soils were extracted with 0.5 M K_2_SO_4_ (1:5 w/v), centrifuged (24,000 *g*, 5 min), and the supernatant recovered for NO_3_^−^ analysis using the vanadate colorimetric procedure of Miranda et al. (2001). The same extracts were used to determine NH_4_^+^ content using the salicylate-based colorimetric procedure of Mulvaney (1996). Dissolved organic C (DOC) and N (DON) in the extracts were measured using an Multi N/C 2100S analyzer (Analytik Jena GmbH, Jena, Germany). CaCO_3_ content was determined with a FOGL Benchtop Soil Calcimeter (BD Inventions, Thessaloniki, Greece). Soluble salts (Na, K, Ca) were determined in soil-distilled water extracts using a Sherwood 410 flame photometer (SciMed Ltd., Stockport, United Kingdom). The rhizosphere soil elemental composition was determined using a non-destructive S2 Picofox TXRF spectrometer (Bruker Inc., Billerica, MA) using Se as an internal standard (Towett et al., 2013). Plant-available P in soil was determined using both the Olsen P method (0.5 M NaHCO_3_ pH 8.5, 1:5 w/v extract) according to FAO (2021) and the acetic acid method (0.5 M CH_3_COOH pH 2.5, 1:5 w/v extract) according to McCray et al. (2012).

### DNA extraction and sequencing

For the DNA extraction, 20 g of soil were washed and resuspended by vortexing with sterile water in a ratio of 1:2 (soil:water). Then, we filtered the supernatant using Millipore Sterivex™ Pressure Filter 0.22 μm and used the filter paper like sample for the DNA extraction. Total DNA was extracted from the samples using E. Z. N. A.® Soil DNA Kit (Omega Bio-tek, Inc.) according to the manufacturer instructions. DNA integrity, quality, and quantity were verified using 1% agarose gel electrophoresis, OD260/280 ratio, and fluorescence using a QubitTM 4.0 Fluorometer along with Qubit dsDNA HS Assay Kit (Thermo Fisher Scientific). For metabarcoding, the total DNA samples were sent to the Environmental Sample Preparation and Sequencing Facility at the Argonne National Laboratory (Illinois, United States) for the PCR amplification of the bacterial 16S rRNA gene V4 hypervariable region [~250 bp; 515F and 806R primers; (Caporaso et al., 2011)], and the construction of paired-end (250 bp) libraries and sequencing on the MiSeq platform (Illumina Inc., San Diego, CA).

### Metabarcoding data processing and analysis

Analyses were conducted in R v3.5.2 and RStudio v1.1.463 (Team, 2023; R Core Team, 2020) using the R package DADA2 v1.16.0 pipeline following https://benjjneb.github.io/dada2/tutorial.html tutorial (Callahan et al., 2016) to infer amplicon sequence variants (ASVs) present in each sample. Briefly, raw reads underwent quality control filtering with the following parameters (no ambiguous bases, Ns = 0; minimum length of 150 bp, maximum expected error rate of 2). Filtered reads were then dereplicated, denoised, and merged into paired-end sequences. After the ASV table was built, chimeras were removed and taxonomic assignment was performed using DADA2’s Ribosomal Database Project’s (RDP) naive Bayesian classifier (Wang et al., 2007) against the Silva v138 database (Quast et al., 2013). The variance-stabilizing transformation was applied to normalize data using the DESeq2 v1.28.1 (Love et al., 2014). A multi-sequence alignment was created using the R package DECIPHER v2.16.1 (Wright, 2016) to infer phylogeny with FastTree v2.1.10 (Price et al., 2009). A phyloseq-object containing the ASVs, taxonomy assignments, phylogenetic tree, and sample metadata was created using the R package Phyloseq v1.32.0 (McMurdie and Holmes, 2013). Alpha diversity indices (Shannon, Simpson, and Chao1) were calculated from this object, while beta diversity (PCoA using Bray Curtis distances) was calculated using the R package ampvis2 v2.4.5 (Andersen et al., 2018).

### Statistical analysis

Differences, assumptions of normality and homogeneity of variance in soil characteristics were determined by a One-way ANOVA test from R project software. Normality of residuals was evaluated using the Shapiro–Wilk test, and homogeneity of variances was assessed using Levene’s test. When assumptions were met, ANOVA followed by a *post-hoc* Tukey’s HSD test was applied to identify differences among sample groups. Tukey *post-hoc* comparison was used when significant differences (*p* < 0.05) were found in each analysis. In addition, a non-parametric Kruskal–Wallis test was conducted to confirm results under a distribution-free approach, and pairwise comparisons were used to identify significant groups. To identify the most relevant physicochemical parameters, an Analysis of Principal Component (PCA) and Redundancy analysis (RDA) was performed using the ampvis2 R package (Andersen et al., 2018).

## Results

### Rhizosphere soil characterization

**Figure 2:**
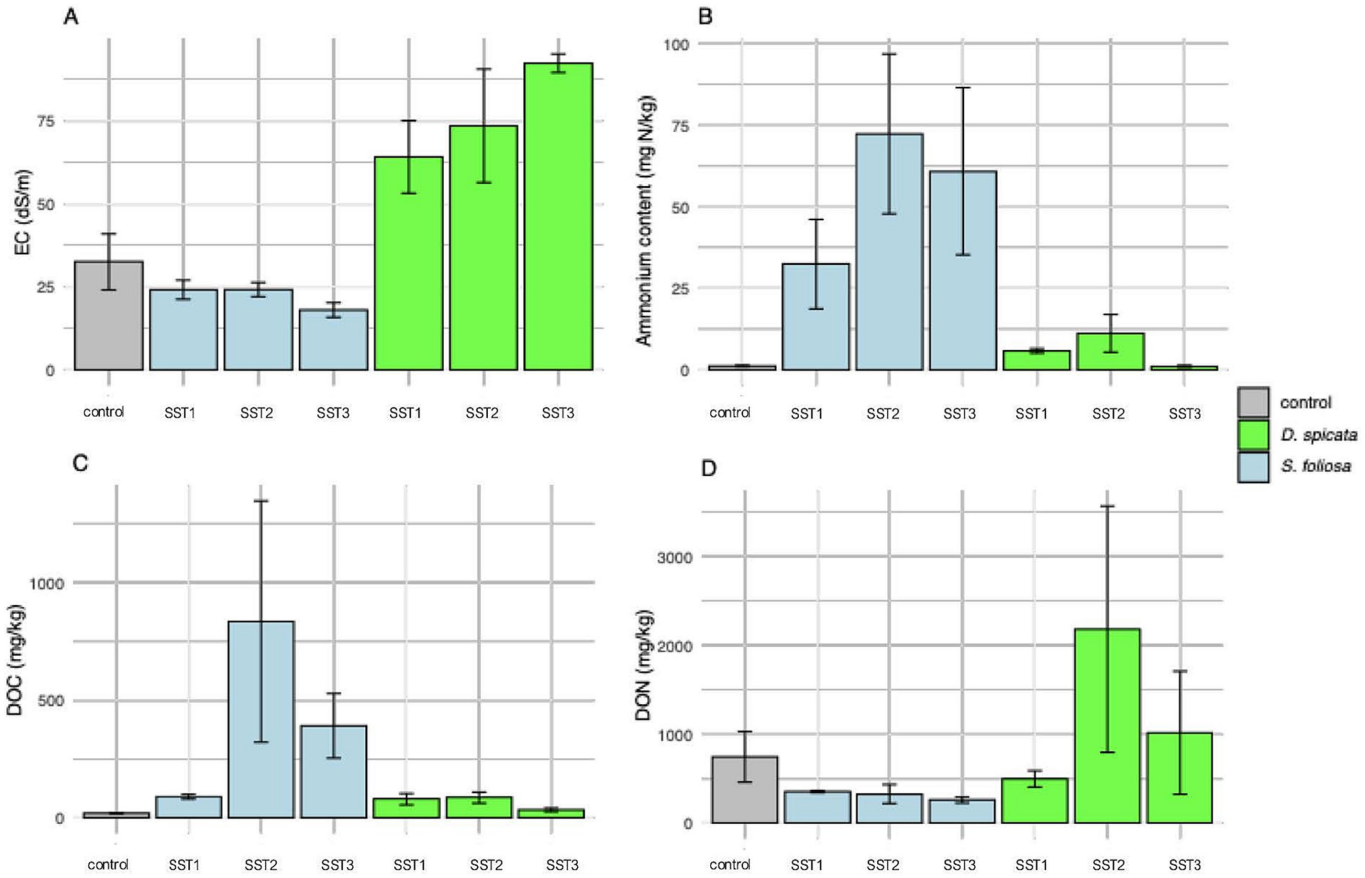
Comparison of physicochemical parameters from *S. foliosa*, *D. spicata* and control unvegetated soil rhizosphere among the sampling stations (SST1-SST3). **(A)** EC, **(B)** Ammonium content, **(C)** Dissolved organic carbon and **(D)** Dissolved organic nitrogen. The soil samples corresponding to Sampling Station indicated in Figure 1A. Values represent means ± SEM (*n* = 5) and statistical differences were assessed using one-way ANOVA followed by Tukey HSD *post-hoc* test. **(A)** *F* = 11.64, *p* > 0.001. **(B)** *F* = 4.16, *p* = 0.013. **(C)** *F* = 4.50, *p* = 0.003. **(D)** *F* = 4.66, *p* = 0.002.

A summary of the physical and chemical properties of rhizosphere soil collected from under the two plants in the hyper-arid core of the Atacama Desert are shown in Supplementary Table 1. The measured pH in our samples was between 6.01 to 7.80, being generally lower under the plants in comparison to the unvegetated control soil. The EC was high in all samples and varied from 18.0 to 92.2 dS/m with values being significatively higher in the *D. spicata* rhizosphere soil in comparison to *S. foliosa* and control soil (Figure 2; Supplementary Table 1). Overall, the concentration of NH_4_^+^ was very low in comparison to NO_3_^−^, however, NH_4_^+^ concentrations were significatively higher in the *S. foliosa* rhizosphere in comparison to the control and *D. spicata* soils (Figure 2). On average, the *S. foliosa* samples soils presented higher NH_4_^+^ contents, and the *D. spicata* rhizosphere soils presented a higher NO_3_^−^ content, although some differences were apparent depending on the sampling station (Supplementary Table 1; Figure 2). Both the Olsen-P and acetic acid-P method recovered very low levels of available P, although more plant-available P was determined in the *S. foliosa* soil using the Olsen-P method. There were no significant differences in plant-available P determined with acetic acid or the CaCO_3_ and moisture content of the soils across the vegetated and control areas (Supplementary Table 1).

### Microbial community composition

**Figure 3:**
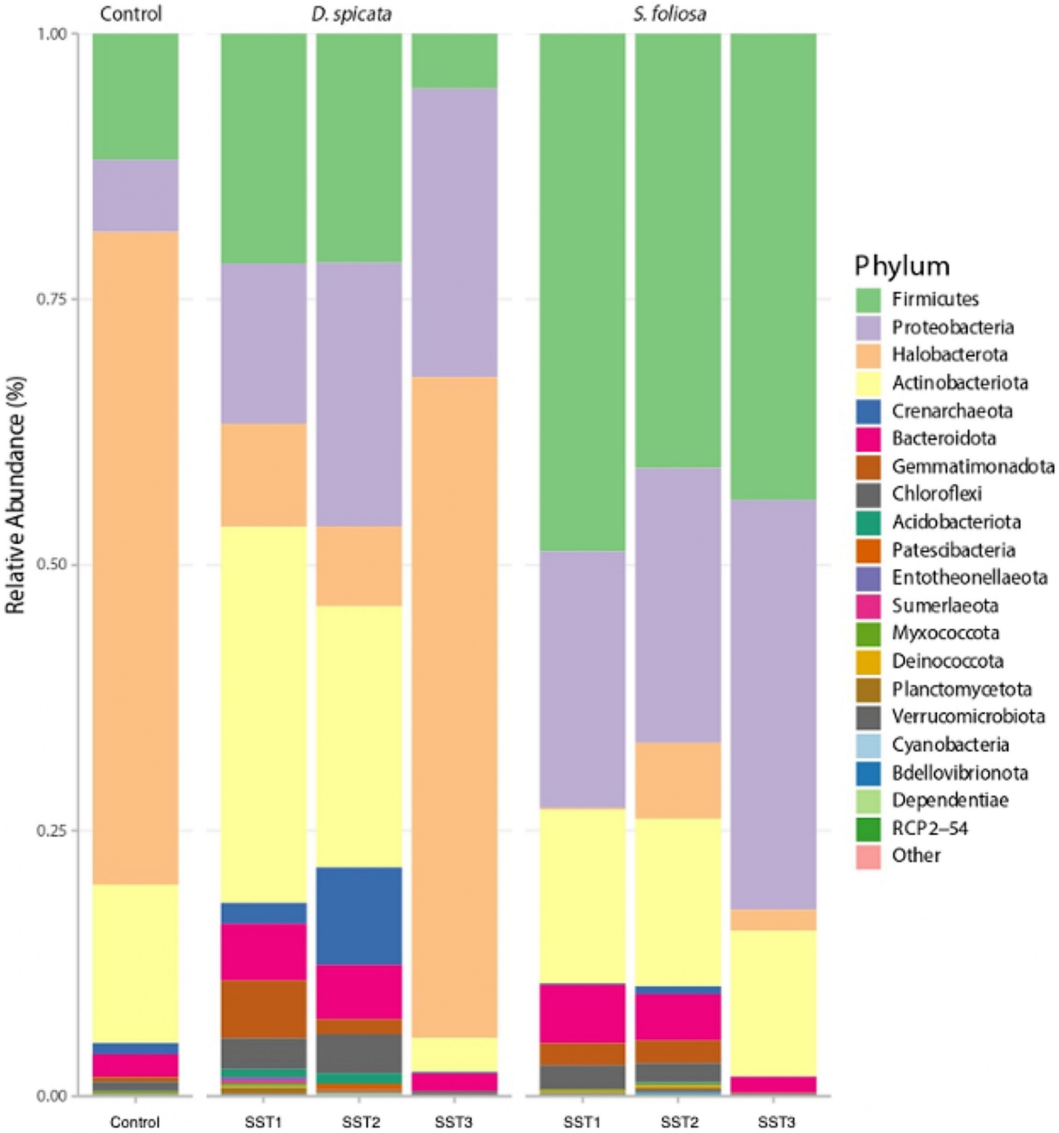
Bacterial community composition at phylum level in the hyper-arid core of the Atacama Desert. Samples include rhizosphere soil from *D. spicata* and *S. foliosa* collected at three sampling stations (SST1–SST3), alongside those collected from control unvegetated soil. Bars represent the mean relative abundance from five replicates per plant species rhizosphere and three replicates for the unvegetated control soils.

Analysis of rhizosphere soil samples revealed distinct bacterial community compositions associated with *D. spicata* and *S. foliosa* in the Yungay Oasis. The 16S rRNA amplicon sequencing identified Firmicutes (Bacillota), Proteobaceria (Pseudomonadota), Halobacteria and Actinobacteriota as the predominant phyla across all soil samples (Figure 3). Halobacteria and Proteobacteria (Pseudomonadota) were the most abundant phyla in the control soils and *D. spicata* rhizosphere, while Firmicutes (Bacillota) and Proteobacteria (Pseudomonadota) were most abundant in the *S. foliosa* rhizosphere. Of note, was the greater abundance of *Halobacteria* in the control soil and *D. spicata* rhizosphere compared to the *S. foliosa* rhizosphere, where Firmicutes (Bacillota) dominated, although this group was also present in *D. spicata* rhizosphere samples (Figure 3).

**Figure 4:**
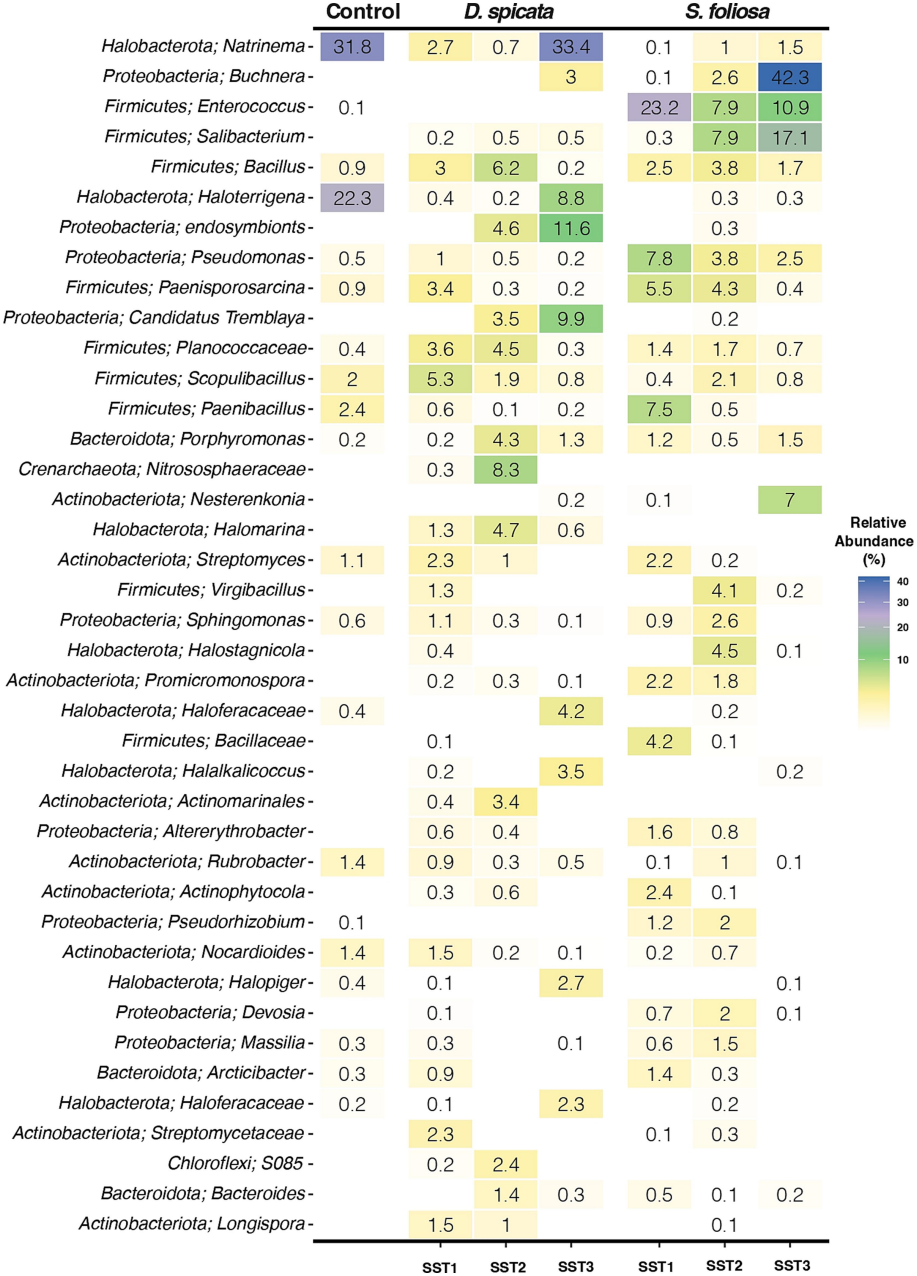
Taxonomic composition and genus abundance of the rhizosphere bacterial community of two native plant species (*D. spicata*, *S. foliosa*) sampled in three sampling stations (SST1-SST3) in the hyper-arid core of the Atacama Desert. Color gradient represents the relative abundance of the top 40 bacterial genera. Figure represents the mean relative abundance from five replicates per plant species rhizosphere and three replicates for the unvegetated control soils.

The 40 most abundant bacterial genera were identified across the three sampling stations to characterize differences between the rhizobiomes of both plant species (Figure 4). In accordance with the results shown above, most of these taxa belong to the Proteobacteria (Pseudomonadota), Firmicutes (Bacillota), Halobacteria and *Actinobacteria* phyla. However, several abundant genera showed substantial variation across sampling stations, with some being highly abundant at certain sampling stations while rare or absent at others, namely: *Natrinema*, *Buchnera*, *Enterococcus* and *Salibacterium*, supporting a high variability in these rhizosphere soil communities. In this context, the variability could be attributed to the extreme conditions of the environment, where microhabitats may differ significantly even within a small area such as native plant rhizosphere. Hence, we hypothesized the native plants in these extreme environments might exhibit low specificity in their microbial associations, potentially forming symbiotic relationships with a broad range of microorganisms that have basic functional requirements to colonize these environments. The results indicate that between *D. spicata* and *S. foliosa* rhizosphere, major differences existed in the relative abundance of the genus *Natrinema* (33.4% vs. 1.5% in SST3), *Buchnera* (3% vs. 42.3% in SST3) and *Enterococcus* (0% in SST1, SST2 and SST3 vs. 23.2, 7.9 and 10.9% for SST1, SST2 and SST3, respectively). Key genera in the control soil and *D. spicata* rhizosphere included: *Natrinema* and *Haloterrigena* with 31.8 and 33.3% for *Natrinema* and 22.3 and 8.8% for *Haloterrigena* in the control and SST3 samples, respectively. Furthermore, in the *S. foliosa* rhizosphere, we found a dominance of the Firmicutes (Bacillota) phylum, specifically for *Buchnera* (42.3%) genus in SST3 and *Enterococcus* genus with 23.2, 7.9 and 10.9% for SST1, SST2 and SST3, respectively. In addition, other Firmicutes (Bacillota) genera such as *Paenisporosarcina* (7.8%) and *Paenibacillus* (7.5%) were also abundant at SST1. SST 3 tended to be more heterogeneous in terms of bacterial taxa abundance and, the control soil was similar to *D. spicata* rhizosphere in taxonomic composition with the presence of genus *Natrinema*, *Haloterrigena*, *Scopulibacillus* and *Nocardioles* (Figure 4).

**Figure 5:**
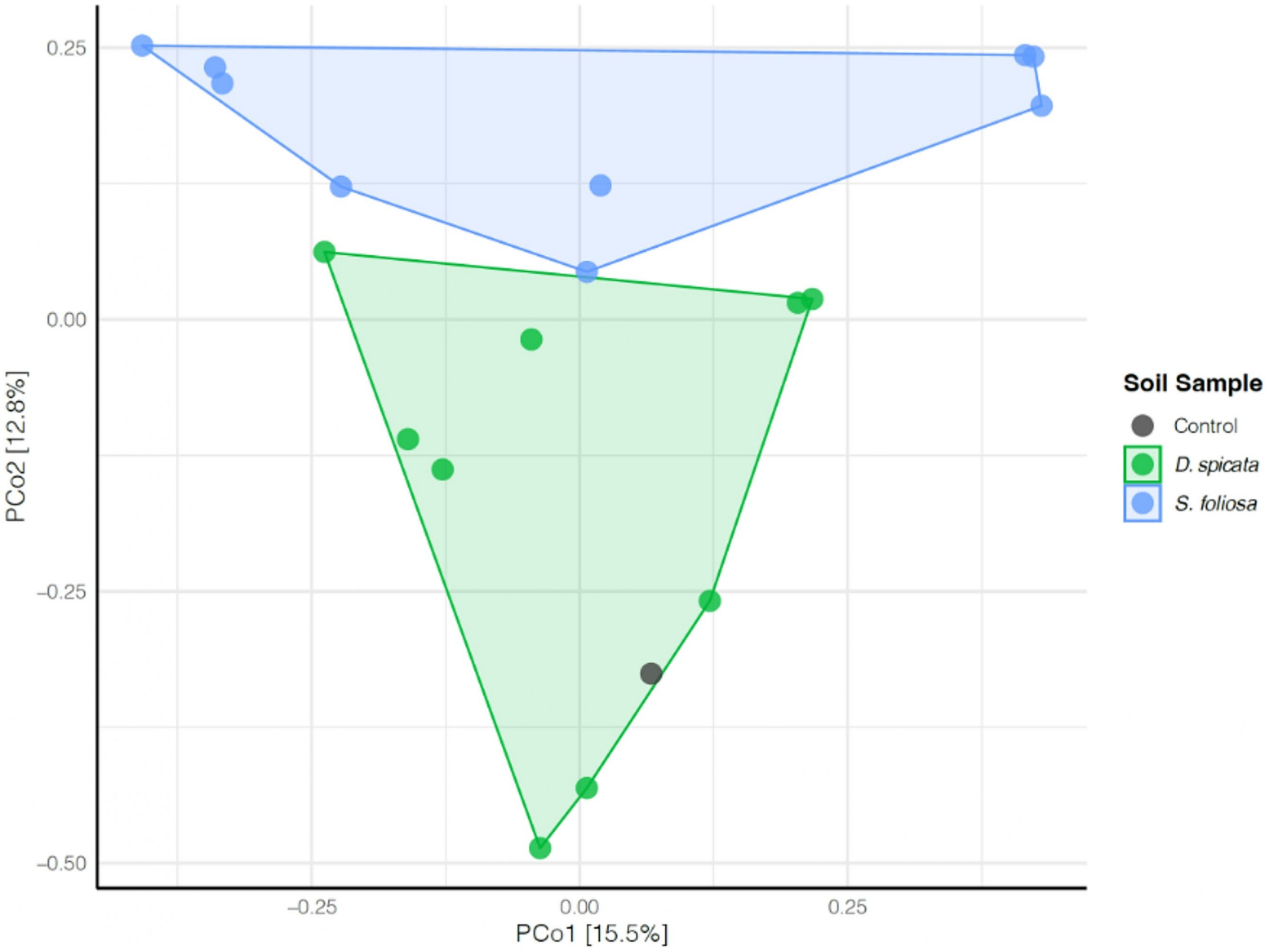
Principal component analysis (PCA) of the rhizosphere bacterial community composition of soil samples collected from under two native plant species (*D. spicata*, *S. foliosa*) alongside control unvegetated soil in the hyper-arid core of the Atacama Desert. The percentage of variance explained is shown on each axis.

Beta diversity analysis revealed distinct bacterial community compositions in the rhizospheres of the two plant species (Figure 5). The bacterial communities from control soils clustered with those of *D. spicata*, while communities associated with *S. foliosa* formed a separate group (Figure 5).

**Figure 6:**
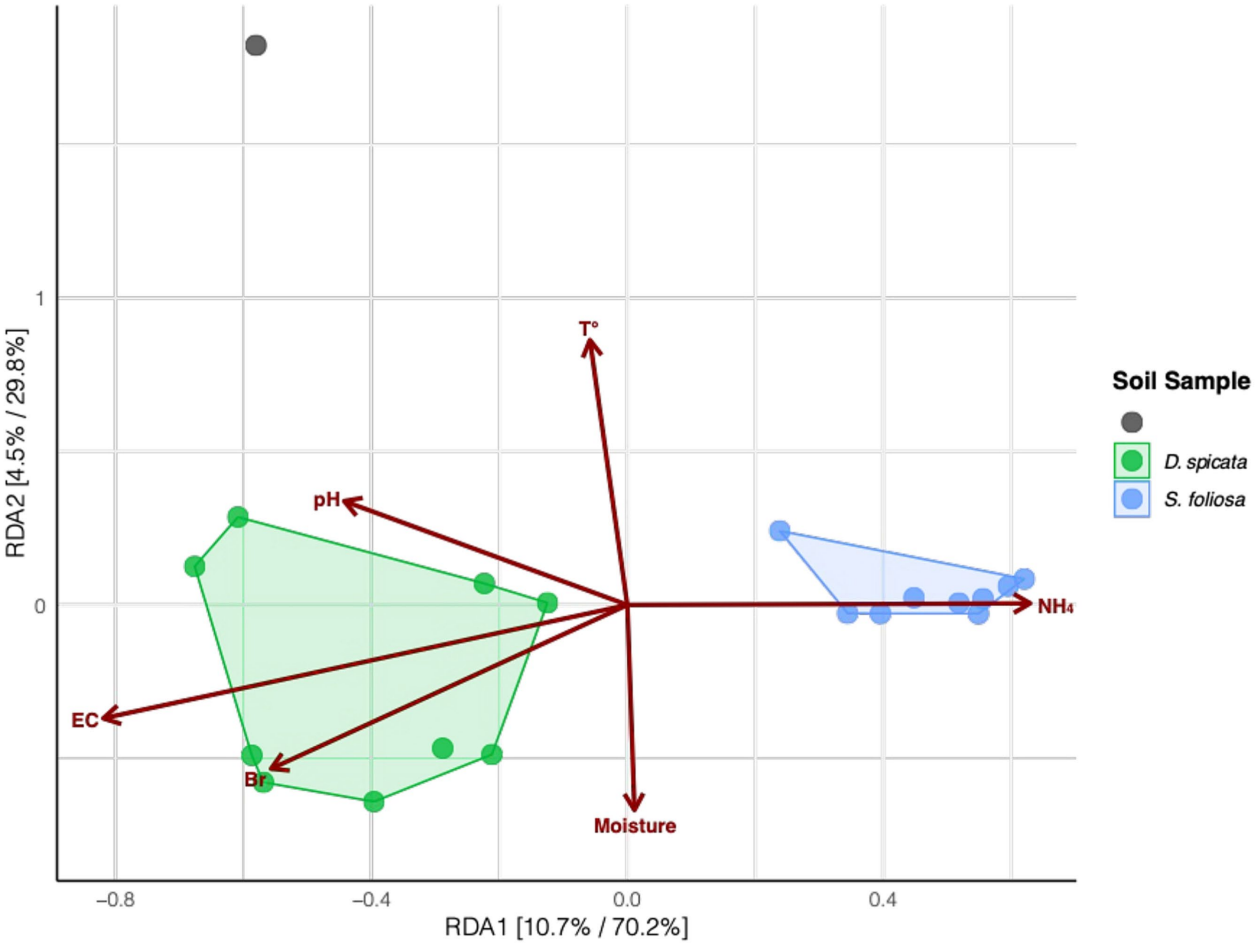
Redundancy analysis (RDA) of rhizosphere bacterial community composition collected from under two native plant species (*D. spicata*, *S. foliosa*) alongside control unvegetated soil in the hyper-arid core of the Atacama Desert. The RDA was based on Hellinger-transformed amplicon sequence variant (ASV) relative abundances. Points represent individual soil samples (colored by plant species for rhizosphere soil, gray for the control soil) with polygons grouping samples by plant species. Arrows indicate the explanatory power of physicochemical parameters in describing the observed variation in bacterial community composition. Percentages on axes show explained variance in the unconstrained and constrained analyses.

We also tested to what extent the measured soil properties could help explain the observed segregation in the microbial communities for the two plant species. Our results showed that the variation was related to several physicochemical parameters, including NH_4_^+^ content, pH and EC. Higher levels of Br, pH and EC were associated with microbial communities from *D. spicata* rhizosphere according to the RDA1 axis, and NH_4_^+^ content were clearly associated with microbial communities from *S. foliosa* (Figure 6; Supplementary Table 2).

## Discussion

### Environmental conditions in the hyper-arid of the Atacama Desert

In this study, we characterized the rhizosphere microbial community associated with *D. spicata* and *S. foliosa* which are located in the hyper-arid core of the Atacama Desert, one of the most extreme environments on Earth. The climatic factors, mainly low precipitation, high day-night temperature regimes and UV irradiation, limit biological productivity and activity, chemical reactions and weathering process (Plaza et al., 2018). Further, these hyper-arid soils possess a low soil organic C content, low microbial activity, and low rates of mineral weathering (Ewing et al., 2006; Knief et al., 2020; Mörchen et al., 2019). However, in this study, we show that it is possible to find an abundance of microorganisms even in hyper-arid soils, specifically in the rhizosphere from native plant species such as *D. spicata* and *S. foliosa*. The differences in soil physicochemical parameters in the rhizosphere soil in comparison to the unvegetated control site, alongside shifts in microbial community composition, strongly suggest that the plants modulate their environment, as described in other sites of the Atacama Desert associated to flowering desert phenomena in the north of Chile (Araya et al., 2020). Our three replicate sample stations (SST1, SST2 and SST3) showed significant variations in NH_4_^+^, NO_3_^−^, Olsen-P, pH, EC, and soluble C (Supplementary Table 1), while CaCO_3_ and moisture content showed no difference (Supplementary Table 1). According to the study area, the wet layers below the surface of the hyper arid core of the Atacama have been reported (Azua-Bustos et al., 2020). It should be noted that we sampled the rhizospheric soil (20–25 cm depth) and were not able to sample the deep soil layers close to the water table. Although the origin of water is outside the scope of this study, the deep-water sources could be related to the unusual rain events as have been indicated by Azua-Bustos et al. (2020). Precipitation events could lead to a reload of a deep groundwater system present in the Aguas Blancas basin (Dirección General de Aguas, 2017). It has a low recharge rate, limited to very sporadic and rare rainfall events (Herrera and Custodio, 2014). Previous reports have shown that the hyper-arid core of the Atacama Desert experiences large day-night variations in air temperature and relative humidity (Azua-Bustos et al., 2015). When combined with low amounts of rainfall, soil moisture, soil surface compaction, high salinity and nutrient imbalance it makes it a highly challenging environment for plants to survive (Marquet et al., 1998). Despite these environmental conditions, we can find dominant plants in the Yungay oasis such as the salty grass *D. spicata*, which has a water-efficient C4 metabolism, is hyper-salt tolerant can survive at >0.5 M NaCl and, can withstand temperatures of up to 57 °C (Golden et al., 1995; Lazarus et al., 2011). The drought-tolerant shrub, *S. foliosa*, with C3 metabolism represents the other dominant higher plant species in the Yungay Oasis (Akhani et al., 1997; Nakano and Asada, 1981) and, has previously been used for the phytoremediation of salt-affected soils (e.g., high Valley of Cochabamba in Bolivia; de Froidmont, 2018).

### Microbial communities associated with native plants in the hyper-arid core of the Atacama Desert

The microbial community in the rhizosphere soil was dominated by Firmicutes (Bacillota), Proteobacteria (Pseudomonadota), Halobacteria and Actinobacteria (Figure 3). However, the microbial community composition may vary in different situations, for example, if we compared the soil surface with subsoil layers, or the interaction of other soil fractions with the rhizosphere is analyzed (Castro-Severyn et al., 2024; Crits-Christoph et al., 2016; Fuentes et al., 2022a; Warren-Rhodes et al., 2019). Our results indicate that the taxonomic composition of *D. spicata* rhizospheric soil exhibited more variability compared to *S. foliosa,* which accounts for a more stable or selected community. In this context, some taxa were found to be very abundant at some sampling sites while being completely absent or having very low representation at others (Figure 4). Similar patterns have been reported in arid environments such as Atacama and North China Desert, which showed high variability in their soil microbial communities (Vásquez-Dean et al., 2020). Additionally, studies in the Namib Desert have reported differences in the microbial communities associated with the roots of *Tetraena simplex*, *Tetraena stapffi* and *Stipagrostis* sp. (Lopes et al., 2024). This may be related to the plant’s need to generate interaction with more specialized organisms capable of tolerating the high salinity or pH changes induced in the soil, which are caused by the plant’s physiologic processes to eliminate salts and thus thrive under desert conditions (Pelliza et al., 2005; Conticello et al., 2002). Under salt stress, certain adapted crops show enhanced root performance (Vaseva et al., 2021). This adaptation is supported by alterations in plant processes (e.g., Na^+^/K^+^ transporter selectivity) as well as microbial mechanisms such as the production of compounds such as indole acetic acid or the enhancement of antioxidant enzyme activity, which can help the plant tolerate stress conditions (Fortt et al., 2022; Khalid and Aftab, 2020; Zhao et al., 2021). Our analysis revealed dominance by the Proteobacteria (Pseudomonadota), Firmicutes (Bacillota), Halobacteria and Actinobacteria phyla. At the genus level, significant differences between the *D. spicata* and *S. foliosa* rhizosphere communities were observed for *Natrinema*, *Buchnera*, *Enterococcus* and *Salibacterium* (Figure 4). Similar patterns of microbial distribution have been observed in other extreme environments, where genera like *Natrinema* and *Haloterrigena* are known to dominate hypersaline soils due to their ability to thrive in high-salinity environments, like saline lakes or salt pans (Crognale et al., 2013; Jiang et al., 2012; Mirete et al., 2015). These genera have been consistently associated with saline environments, suggesting that their presence in the *D. spicata* rhizosphere could be directly related to the high salt concentrations observed in these soils (Jones et al., 2023; Remonsellez et al., 2018). Furthermore, *Buchnera* and *Enterococcus*, typically associated with gut symbiosis, have also been found in plant environments, where their presence may be explained by their role in N cycling or plant growth promotion in stressful conditions (Bartelme et al., 2018; Zhang et al., 2020). Moreover, the dominance of Firmicutes (Bacillota) in *S. foliosa* rhizosphere, particularly *Enterococcus* and *Buchnera*, could be an adaptive response to the ammonium-rich environment, as seen in other studies where these genera have thrived in nutrient-stressed ecosystems related to phosphate limitation (Knief et al., 2020; Neilson et al., 2017). We ascribe the intrinsic variability observed in microbial composition between sampling sites to microenvironmental differences, as extreme conditions in the Atacama Desert are typically highly heterogeneous even within small areas (Crits-Christoph et al., 2016). Also, the assembly mechanisms and ecosystem functions of hypolithic communities from the Qaidam Desert in China, which occupy highly specialized microenvironments beneath translucent rocks in arid environments, are not yet fully understood (Lai et al., 2024). As well as a study from Chihuahuan Desert in Mexico related to plant-bacteria interaction, specifically the promote facilitation from nurse plant relationship have been reported that facilitation is enhanced by plant species that provide a more homogeneous microenvironment (Sánchez-Martín et al., 2024).

Other studies demonstrated that *Actinobacteria* and *Chloroflexi* dominate soil microbial communities in the hyper-arid margin of the Atacama Desert with vegetable cover (Neilson et al., 2012, 2017). Furthermore, most recent studies showed the dominance of different groups of Actinobacteria, Proteobacteria and Chloroflexi in Atacama Desert soils (Knief et al., 2020), while Connon et al. (2007) detected Actinobacteria, Proteobacteria (Pseudomonadota) and Firmicutes (Bacillota) in Yungay soil (0–20 cm depth), similar to our findings. Our analysis revealed a strong correlation between EC and the abundance of *Halobacteria* in the *D. spicata* rhizosphere, reflecting the relationship between soil salinity and halophilic bacterial communities. Furthermore, the higher ammonium content in the *S. foliosa* rhizosphere could favor *Firmicutes* phyla, although they are not commonly classed as ammoniaphilic and, the high Br content may be related to salt excretion by *D. spicata* and the geochemical composition of the surrounding soil (Morris et al., 2019; Figure 6). Soil moisture is essential for bacterial diversity in desert soils (Bottos et al., 2020), but in this work the moisture does not present statistical differences between the rhizosphere of two plant species, perhaps because in 2022 the Yungay area experienced a slight episode of precipitation and fog (Weather and Climate, 2022). Castro-Severyn et al. (2024) demonstrated that rhizosphere soil enrichment cultures from *S. foliosa* and *D. spicata* were dominated by *Klebsiella*, followed by *Brevibacillus,* reflecting the selection patterns at the phylum level Proteobacteria (Pseudomonadota) and Firmicutes (Bacillota), respectively for both plant species. In this context, different species of the *Klebsiella* genus have been repeatedly detected in many soil types and environmental samples, including soils from the Yungay area (Ekwanzala et al., 2019; Thomas et al., 2006). The soil moisture could be ancillary to other factors in shaping bacterial diversity and, the clay-rich soils can act as a possible “water reservoir” and help shape microbial life conditions (Fuentes et al., 2022a). Moreover, the salinity of the soil, reflected by the EC, was high in soil from the *D. spicata* rhizosphere (Figure 2A), and it seems to shape bacterial communities due to intense selective pressure, as few bacteria are capable of growing over large gradients of salt concentrations (Figures 3, 5). We measured high EC values (over 64 to 92 dS/m) in *D. spicata* rhizosphere, while in *S. foliosa* rhizosphere, the EC values fluctuated between 18 and 24 dS/m (Supplementary Table 1). In this regard, we observed a particular accumulation of halophilic taxa in the *D. spicata* rhizosphere; specifically, in samples from Sampling Station 3. This halophytic trait is reflected in the high contents of NaCl in the leaves, the salt crystals presence on the leaf surface in *D. spicata* and the presence of high amounts of salt in the phyllosphere soil (Jones et al., 2023). In the same work, Jones et al. (2023) presented a comparison between vegetated and unvegetated soils, suggesting that *D. spicata* is effective at removing salt from the soil and translocating it to above-ground component (e.g., much lower levels of Na were seen in the roots relative to the shoots and in the subsoil relative to the phyllosphere soil). These observations are consistent with the foliar Na excretion through salt glands in this plant, as reported in other studies (Hasanuzzaman et al., 2014; Semenova et al., 2010). Furthermore, it is interesting to note that some of the bacterial families present in the soil profile are known to include halophilic taxa or have been shown to be present in other saline environments, such as Proteobacteria (Pseudomonadota), Actinobacteria, and Firmicutes (Bacillota) which have been detected in different saline environments (Bartelme et al., 2018; Crognale et al., 2013; Jiang et al., 2012; Mirete et al., 2015; Remonsellez et al., 2018; Zhang et al., 2020).

### Ecology importance of *S. foliosa* and *D. spicata* microbial communities

To date, only one study has reported NH_3_ emissions occurring during the microbial processing of organic-N in the Atacama Desert (Jones et al., 2023), this information can explain the high levels of nitrogen compounds (TN and NO_3_^−^) in our samples from *D. spicata*. Furthermore, different studies have reported an association between microbial richness and diversity in the Atacama Desert hyper-arid soils with water availability, relative humidity and TC content (Crits-Christoph et al., 2016; Fuentes et al., 2022a; Knief et al., 2020; Neilson et al., 2017; Schulze-Makuch et al., 2018). Furthermore, it would be important to highlight the functional potential of the microbial community associated with *D. spicata* and *S. foliosa*. In this context, there could be related physiological functions such as salt excretion in *D. spicata* plants (Yuan et al., 2016; Morris et al., 2019) with microbial functions such as osmolytes biosynthesis. Moreover, the high ammonium content in the *S. foliosa* rhizosphere could be an ammonium accumulation under stress conditions (Rütting et al., 2011). In this respect, other studies have reported microbial functions related to nitrate and nitrite ammonification in plants from arid environments (Kuypers et al., 2018). Despite this knowledge, to date there has been no metagenomic data relating to microbial community functions and plant-bacteria interactions from soils of the hyper-arid core of the Atacama Desert. Therefore, the search for life in extreme environments should consider bacterial, archaea and fungi communities functioning to better understand how native plants thrive under the combined abiotic stresses operating in the Atacama Desert.

## Conclusion

The hyper-arid core of the Atacama Desert represents one of the driest ecosystems on Earth, while the prevailing environmental conditions make it one of the most extreme places for life to establish. In this study, we characterized the rhizosphere bacterial communities and soil properties associated with two native plant species (*D. spicata* and *S. foliosa*) in the Yungay Oasis. In this context, it is important to remark that to date no studies related to plant-bacteria interaction in this area from the hyper arid core of the Atacama Desert. Our analyses revealed distinct microbial community structures correlating with specific soil parameters, particularly EC and ammonium content. This study advances our fundamental understanding of plant-microbe interactions in extreme environments by identifying bacterial communities capable of thriving in the hyper-arid core of the Atacama Desert and their community structure depends on the soil parameters and plant interaction in the rhizospheric soil environment. Future research should focus on functional studies by metagenomic approach to elucidate the specific roles of these microbial communities in plant-bacteria interactions within these extreme soils.

## Data Availability

The original contributions presented in the study are publicly available. This data can be found at: https://www.ncbi.nlm.nih.gov/bioproject/, accession number PRJNA971922.
