# Supplementary material for: Plant species-specific rhizobiome assembly in the hyper-arid Atacama Desert
Source: Front Microbiol. 2025 Sep 16;16:1587491. doi: 10.3389/fmicb.2025.1587491 (PMC12479509; doi:10.3389/fmicb.2025.1587491)
Supplement: Supplementary file 1 [file Supplementary_file_1.docx]

*Supplementary Material*

*Supplementary data*

***
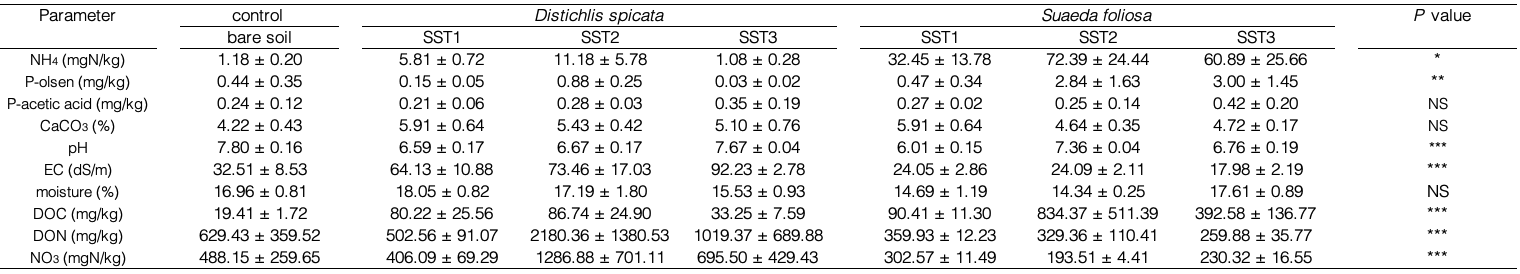
***

**Supplementary table 1.** Summary of physicochemical parameters from all soil samples analyzed in this work. Values represent means ± SEM (*n* = 33). Significance differences for each treatment are P < 0.05 level. The P value ANOVA symbols *, ** and *** indicate significant differences at the P < 0.05, P < 0.01 and P < 0.001 level, respectively, while NS indicate no significant difference (P > 0.05). SST1-SST3 represent the three replicate sampling areas.

**Supplementary Table 2 (Part 1).** Physicochemical parameters of 33 soil samples from the Yungay oasis, element values from TXRF assay are listed in µg/g.

**Supplementary Table 2 (Part 2).** Physicochemical parameters of 33 soil samples from the Yungay oasis, element values from TXRF assay are listed in µg/g.
